# Inactivation of Hepatitis A Virus and Human Norovirus in Clams Subjected to Heat Treatment

**DOI:** 10.3389/fmicb.2020.578328

**Published:** 2021-01-12

**Authors:** Cristina Fuentes, Francisco J. Pérez-Rodríguez, Aurora Sabrià, Nerea Beguiristain, Rosa M. Pintó, Susana Guix, Albert Bosch

**Affiliations:** ^1^Enteric Virus Laboratory, Department of Genetics, Microbiology and Statistics, University of Barcelona, Barcelona, Spain; ^2^Nutrition and Food Safety Research Institute (INSA⋅UB), University of Barcelona, Barcelona, Spain

**Keywords:** hepatitis A virus, human norovirus, clams, heat inactivation, infectivity, PMA-viability RTqPCR

## Abstract

Bivalve mollusk contamination by enteric viruses, especially human noroviruses (HuNoV) and hepatitis A virus (HAV), is a problem with health and economic implications. The aim of the study was the evaluation of the effect of heat treatment in clams (*Tawera gayi*) experimentally contaminated with HuNoV using a PMA-viability RTqPCR assay to minimize measurement of non-infectious viruses, and used HAV as a model to estimate infectivity loss. Spiked clams were immersed in water at 90°C to ensure that internal meat temperature was maintained above 90°C for at least 5 min. The treatment resulted in >3.89 ± 0.24 log_10_ TCID_50_/g reduction of infectious HAV, confirming inactivation. For HuNoV, RTqPCR assays showed log_10_ reductions of 2.96 ± 0.79 and 2.56 ± 0.56, for GI and GII, respectively, and the use of PMA resulted in an additional log_10_ reduction for GII, providing a better correlation with risk reduction. In the absence of a cell culture system which could be used to determine HuNoV infectivity reduction, a performance criteria based on PMA-RTqPCR log reduction could be used to evaluate food product safety. According to data from this study, heat treatments of clams which cause reductions >3.5 log_10_ for GII as measured by PMA-RTqPCR assay may be regarded as an acceptable inactivation treatment, and could be set as a performance criterion to test the effectiveness of other time-temperature inactivation processes.

## Introduction

The occurrence of gastroenteritis due to human norovirus (HuNoV) and hepatitis a virus (HAV) infections associated to the consumption of contaminated mollusks is a major public health concern. The analysis of the European baseline survey of HuNoV in oysters reported a prevalence of 34.5% in production areas (Romalde et al., 2018). Although surveillance studies report lower prevalences for HAV in bivalves produced within the EU (Romalde et al., 2018; Fusco et al., 2019), HAV outbreaks have occurred in association to consumption of both local (Guillois-Bécel et al., 2009; Boxman et al., 2016) and imported mollusks (Pinto et al., 2009). According to data from the European Union (EU) in 2018, enteric viruses generated 389 foodborne outbreaks affecting approximately 10,000 individuals, and HuNoV accounted for 20% of all outbreaks associated to fish and fishery products including shellfish and mollusks (Fusco et al., 2019).

The most reliable virucidal method to ensure mollusk safety is thorough cooking. Heat-treatments ensuring that pathogenic microorganisms are eliminated must also be industrially applied by shellfish producers to fulfill current EU regulations for samples from Class B and C production areas which do not comply with bacteriological criteria. According to EU official classification scheme, mollusk production areas are classified as Class B when at ≥90% of samples contain less than 4,600 *E. coli* most probable number (MPN) per 100 g of flesh and intravalvular fluid and remaining 10% below 46,000; and as Class C when all samples have <46,000 *E. coli* MPN/100 g. The heat treatment of reference is the heating of the mollusk flesh to not less than 90°C and maintenance of this minimum temperature for a period of not less than 90 s (Codex, 2012), but based on studies performed with HAV, it is known that heat-up and cool-down time can lead to significant variations in viral log_10_ reduction depending on the process design (EFSA BIOHAZ Panel, 2015). A heat inactivation and risk assessment model elaborated by EFSA, based on HAV data, highlighted the need for a Performance Criterion (PC) for the whole process, which is the required log reduction during heat treatment (EFSA BIOHAZ Panel, 2015). While in the absence of heat treatment, the average predicted risk was about 1 infection per 100 servings, 4 logs reduction of HAV during heat inactivation would reduce the predicted risk down to one infection per 1,000,000 servings. For HuNoV, despite availability of a cell culture systems to propagate certain HuNoV (Ettayebi et al., 2016) strains, its application for viral inactivation studies on food matrixes is still difficult and data on inactivation kinetics have been mostly estimated by measuring genome copies only and by the use of viral surrogates (Bozkurt et al., 2015; Polo et al., 2018). Both approaches have generated data that suggest that HuNoV is less tolerable to heating than HAV (Bozkurt et al., 2015). As an example, D-values at 100°C for HAV and HuNoV estimated by reduction in viral genome copies in contaminated mussels are 1.58 min and 0.93–1.3 min, respectively (Hewitt and Greening, 2006; Croci et al., 2012; Bozkurt et al., 2015).

Several inactivation studies related to food safety issues have also been addressed recently by using PMA-viability RTqPCR assays, but the number of reports on the applicability of such methods on complex matrixes such as bivalve mollusks are still low (Moreno et al., 2015; Quijada et al., 2016; Randazzo et al., 2018).

The objective of the present work was to evaluate the effect of heat treatment in mollusks experimentally contaminated with HuNoV and HAV, inferred by PMA-viability RTqPCR assay to minimize measurement of non-infectious viruses. Clams were selected due to their high reported levels of HuNoV contamination (Guix et al., 2019), because they are often heat-treated by producers and/or distributors and sold as cooked, and due to the lack of published studies focusing on HuNoV inactivation in this type of mollusk. Heat treatment was designed so that it could be assured that internal temperature of clams was maintained above 90°C for at least 90 s, following the EU regulatory guidelines, and seeking a performance criterion of at least 4 log_10_ inactivation of infectious viruses (Millard et al., 1987; Lees, 2000). The generated data will be valuable to improve the thermal inactivation models existing for HAV and to contribute to the better understanding of HuNoV heat tolerability in shellfish.

## Materials and Methods

### Viral Stocks

Stool specimens positive for HuNoV GI.6 and GII.4 (New_Orleans_2009) from patients with gastroenteritis were obtained from previously studied gastroenteritis outbreaks (Sabria et al., 2014). Ten percentage (wt/v) stool suspensions in phosphate buffered saline (PBS) buffer were prepared and viral genome concentrations were determined by RTqPCR quantification, following the protocol described at the ISO 15216-1:2017 (ISO15216-1:2017,2017), as previously described (Boxman et al., 2016). The cell-adapted cytopathogenic pHM175 43c strain of HAV (kindly provided by T. Cromeans, Centers for Disease Control and Prevention, Atlanta, GA) was grown in FRhK-4 cells and concentrated virus stocks were obtained as previously described (Aragonès et al., 2008). Viral stocks were titrated by Tissue Culture Infectious Dose 50 (TCID_50_) assay in FRhK-4 cells, as well as by RTqPCR quantification following the protocol described at the ISO 15216-1:2017 (ISO15216-1:2017,2017).

### Artificial Contamination of Clams

Clams (*Tawera gayi*) from two batches which had been previously tested negative for HuNoV and HAV by ISO 15216-1:2017 (ISO15216-1:2017,2017) method, were kindly provided by Mascato SA (Vigo, Spain). A pool of HuNoV GI, GII, and HAV viruses was prepared at a final concentration of 2.59 × 10^7^, 4.25 × 10^8^, and 2.12 × 10^11^ viral genome copies per ml, respectively (the concentration of HAV in TCID_50_/ml was of 6.69 × 10^9^). Clams were spiked by manually injecting 10 μl of the virus pool into the hepatopancreas of each animal using a 10-μl syringe with a very fine needle. Viruses were left to adsorb to the digestive tissue for 120 min at room temperature, and contaminated clams were vacuum packed in 15 × 15 cm plastic bags. Twelve clams from different batches were included in each bag per condition.

### Thermal Inactivation

Contaminated clams vacuum packed in 15 × 15 cm plastic bags were processed by thermal treatment in a water bath at 90°C. Samples were immersed in the water bath for 10 min to ensure that samples reached 90°C for at least 90 sec. On average, 4 min were required for samples to reach 90°C and temperature was above 90°C during 5 min. Temperature within each vacuum packed bag was monitored using a digital thermograph (Datalogger EBI10). After treatment, packed clams were transferred to water at room temperature to reduce temperature, and were further frozen at −70°C until processing. Each condition was replicated three times.

### Sample Processing and Virus Quantification

Digestive glands were dissected out from the 12 contaminated clams contained in each bag and were manually chopped with a razor blade to a paste-like consistency. Approximately 2 g digestive tissue (DT) samples were obtained from the 12 clams contained in each bag. The obtained DT samples were split in two parts to be used in the RTqPCR (HuNoV GI, GII, and HAV) and infectivity assays (HAV), respectively.

For RTqPCR assays, the DT mass was diluted at a 1:1.5 proportion in proteinase K solution (3 U/ml). Ten microliteres of a process control Mengovirus stock were added to each sample and they were digested for 1 h at 37°C and 15 min at 60°C. The suspension was centrifuged at 800 g for 5 min at 4°C, the pellet was discarded, and this was considered the DT homogenate. 500 μl of the DT homogenate were processed using the NucliSens^®^ miniMAG magnetic kit (BioMérieux) according to the manufacturer’s instructions. Nucleic acids were eluted in 100 μl and RTqPCR quantification was performed following the protocol described at the ISO 15216-1:2017 (ISO15216-1:2017,2017). Limit of detection (LOD) of the RTqPCR analysis of HAV, HuNoV GI and HuNoV GII on shellfish using the ISO 15216-1:2017 was of 198, 34 and 53 Genome copies/g DT, respectively (Lowther et al., 2019).

For PMA-viability RTqPCR assays, 50 μl of the DT homogenate were diluted 1:10 in a final volume of 500 μl of distilled water containing 50 μM of PMA (Biotinum). The mixtures were incubated in the dark for 5 min at 300 rpm, and were then exposed to light for 15 min using a photo activation system (Led-Active Blue, Geniul). Subsequently, nucleic acid extraction and RTqPCR quantification was performed following the protocol described at the ISO 15216-1:2017 (ISO15216-1:2017,2017).

For determination of infectious viral titer, the DT mass was mixed with GBEB (Glycine Beef Extract Buffer: 1.5% beef extract in glycine 0.05 M) pH 9.5 at a proportion 1:3, and incubated at room temperature for 20 min at 30 rpm. The suspension was centrifuged at 800 g for 15 min at 4°C and the pellet was discarded. The supernatant was diluted 1:1 with Minimal Essential Medium (MEM) and the pH was adjusted at 7 ± 0.5. The sample was decontaminated by the addition of chloroform at 30% (v/v) and vigorous mixing. The aqueous phase was recovered by centrifugation at 9,300 g for 10 min at room temperature. Chloroform traces were removed through aeration of the supernatant, and the final recovered volume was measured. Infectious HAV titers were determined using the TCID_50_ method in FRhK-4 cells (Manso and Romalde, 2013), testing a total volume of 1.6 ml for each sample. All samples were titrated in duplicate.

### Data Analysis

Viral recoveries were calculated by dividing the virus recovered from each sample (bag containing 12 clams) by the total amount of viruses inoculated in each sample, using the formula % Recovery = Measured Recovered Virus Concentration/(Total Viruses Spiked in 12 clams/Total DT g obtained from 12 clams) × 100. Effect of heat treatment on virus reduction was determined by calculating the log reduction units (N_*t*_/N_0_), where N_0_ is the titer of virus recovered on untreated control samples and N_*t*_ is the titer of virus recovered on heat inactivated samples. Differences between groups were determined by one-way analysis of variance (ANOVA) and HSD Tukey’s test, using the VassarStats website^1^. A *p*-value < 0.05 was deemed significant.

## Results

The efficiency of virus extraction procedures from experimentally spiked clams were calculated using unheated samples and are shown in Table 1. While efficiencies for both HuNoV genogroups were above 1%, efficiencies for HAV were below 1% both by measuring infectious viruses as well as viral genomes. In all cases, recoveries of Mengovirus process control were above 1% (3.21 ± 0.18%) as required in the ISO 15216-1:2017 (ISO15216-1:2017,2017), and RTqPCR inhibition was not detected in any sample.

**TABLE 1:** Recovery efficiencies of spiked viruses (average ± standard deviation).

Viral target	Spiked	Recovered	% Recovery^a^
*(a) TCID_50_ assay^b^*			
HAV	8.60 ± 0.07	5.75 ± 0.32	0.16 ± 0.11
*(b) RTqPCR assay^c,d^*			
HAV	10.11 ± 0.07	7.32 ± 0.40	0.24 ± 0.27^AB^
HuNoV GI	6.19 ± 0.07	4.18 ± 0.26	1.17 ± 0.87^ABC^
HuNoV GII	7.33 ± 0.05	5.62 ± 0.19	2.05 ± 0.78^C^

*^*a*^Recoveries for each sample (bag containing 12 clams) were calculated using the formula % Recovery = Measured Recovered Virus Concentration/(Total Viruses Spiked in 12 clams/Total DT g obtained from 12 clams) × *100. Titers were expressed as TCID_50_ or Genome copies/g DT. As an example for HAV measured by TCID_50_ assay, 12 clams from one sample were inoculated with a total of 7.93 × 10^8^ TCID_50_, and a total of 1.77 g were obtained after dissecting all 12 clams. Since HAV concentration measured after animal DT analysis was 5.50 × 10^5^ TCID_50_/g DT, % recovery was 5.50 × 10^5^/(7.93 × 10^8^/1.77)* × 100 = 0.12%. Table shows mean values ± standard deviations from three samples.^*b*^Spiked and recovered levels are expressed as Log TCID_50_/g DT.^*c*^Spiked and recovered levels are expressed as Log Genome copies/g DT.^*d*^Values with different capital letters in the third column denote significant differences between viruses (p < 0.05).*

**FIGURE 1:**
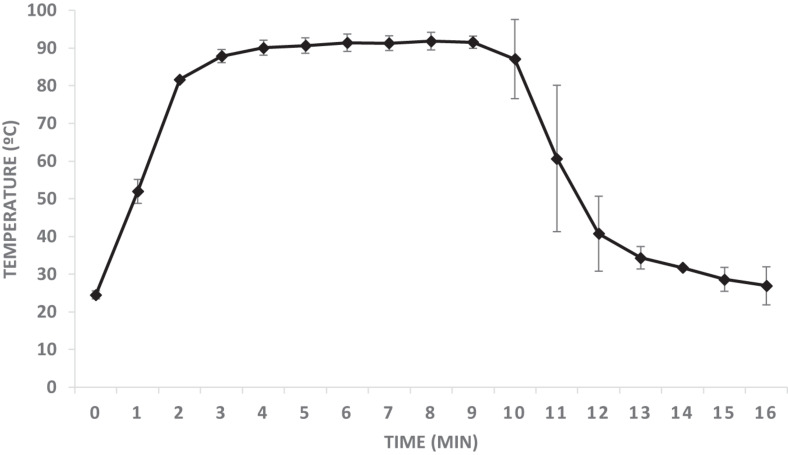
Temperature monitoring during the inactivation process. Data correspond to average ± standard deviation calculated from three independent experiments.

For viral inactivation experiments, spiked clam samples were immersed in a water bath at 90°C within vacuum packed bags, and temperature within each bag was monitored using a thermograph. Figure 1 shows the average temperature recorded during the process. On average, 4 min were required for samples to reach 90°C and temperature was above 90°C during 5 min.

Experiments confirmed a log_10_ reduction of >3.89 ± 0.24 log TCID_50_/g for infectious HAV, confirming that treatment performed caused viral inactivation, and as expected measurement of viral inactivation by RTqPCR assay alone produced a much lower log_10_ reduction (Table 2). For HuNoV, RTqPCR assays showed higher log_10_ reductions than for HAV (2.96 ± 0.79 and 2.56 ± 0.56, for GI and GII, respectively), according to a lower tolerance to heat, but differences were not statistically significant (p = 0.088). Finally, the use of PMA provided a better correlation with infectivity, with additional log_10_ reductions of 1.41 and 0.96, for HAV and GII, respectively. Viral titers for HAV and GII untreated samples measured by RTqPCR and PMA-RTqPCR assays and expressed as Genome copies/g DT were similar. PMA on GI contaminated samples could not be applied due to insufficient viral titers. The lack of signal in the untreated controls after PMA treatment could be due to the fact that a 1/10 dilution of the DT was made before addition of PMA in order to ensure its optimal action, and also be to the occurrence of damaged GI capsids in the stool specimen used for spiking. Since temperature profile was dynamic (Figure 1) and no intermediate data were collected during heat treatment, D values could not be assessed precisely.

**TABLE 2:** Titers and log reductions (average ± standard deviation) for hepatitis A virus (HAV), HuNoV GI, and GII in clams during heat inactivation.

	Untreated samples (N_0_)	Heat-treated samples (N_*t*_)	Log N_*t*_/N_0_
*(a) HAV*			
TCID_50_ assay^a^	5.75 ± 0.32	<1.85 ± 0.02	>3.89 ± 0.24
RTqPCR assay^b^	7.32 ± 0.40	5.57 ± 0.35	−1.75 ± 0.13
PMA-RTqPCR assay^b^	7.79 ± 0.36	4.63 ± 1.21	−3.16 ± 1.46
*(b) HuNoV GI*			
RTqPCR assay^b^	4.18 ± 0.26	1.22 ± 0.59	−2.96 ± 0.79
PMA-RTqPCR assay^b^	<LOD	<LOD	NA
*(c) HuNoV GII*			
RTqPCR assay^b^	5.62 ± 0.19	2.97 ± 0.40	−2.56 ± 0.56
PMA-RTqPCR assay^b^	5.86 ± 0.35	2.34^c^	−3.52^c^

*LOD, Limit of detection; NA, Not available.^*a*^Titers N_*t*_ and N_0_ are expressed as Log TCID_50_/g DT.^*b*^Titers N_*t*_ and N_0_ are expressed as Log Genome copies/g DT.^*c*^Two of the three replicas were < LOD.*

## Discussion

In the present study, heat inactivation of HAV and HuNoV on clams (*Tawera gayi*) was evaluated, by using a heat treatment which assured that internal temperature of clams was maintained above 90°C for at least 90 s, following the EU regulatory guidelines. HAV was used as the most heat-resistant model virus which allow infectivity measurement. Experiments were designed in order to achieve a performance criterion of >4 log_10_ reduction of infectious viruses, but the difficulty in obtaining HAV stocks with extremely high titers allowed us to confirm a log_10_ reduction of >3.89 because the titer fell below the limit of detection of our assay. According to the process lethality determination model made available by the Foundation for Meat and Poultry Research and Education^2^, and the HAV D and z reference values published by EFSA BIOHAZ Panel (2015), the log reduction estimated by the heat treatment performed in our study would be 10.65, ensuring a complete virucidal effect. When assessed by PMA-RTqPCR, a molecular assay which has been shown to allow infectivity estimates for other food matrices (Moreno et al., 2015), this performance corresponded to a log_10_ reduction of 3.16 ± 1.46.

Although inactivation of HuNoV is normally only assessed by molecular methods and/or surrogates, published data indicate that it is less tolerable to heating than HAV (Hewitt and Greening, 2006; Croci et al., 2012; Bozkurt et al., 2015). The Human Intestinal Enteroid (HIE) infection model also demonstrated complete inactivation of GII.3 and GII.4 by heating at 60°C for as little as 15 min (Ettayebi et al., 2016). In our study, both RTqPCR and PMA-RTqPCR measurements confirmed that heat-resistance of HuNoV is lower than HAV.

The use of PMA-RTqPCR viability assays has been previously tested and optimized for HAV and HuNoV, to be applied on complex mollusk samples (Moreno et al., 2015; Randazzo et al., 2018). For HAV, the use of 10-fold diluted cockle or clam DT homogenate and pretreatment with 50 μM PMA combined with 0.5% Triton X-100 allowed the minimization of the detection of spiked thermally inactivated viruses (Moreno et al., 2015). For HuNoV, pretreatment was optimized by using 100 μM PMAxx with 0.5% Triton X-100 and increasing incubation and photoactivation processes, and oysters contaminated by bioaccumulations were used (Randazzo et al., 2018). None of these studies completely eliminated the quantification of genomes despite the performance of heat-treatments which would provide complete inactivation. Our protocol was based on the use of 10-fold diluted clam DT homogenate coupled with 50 μM PMA. It allowed additional log_10_ reductions of 1.41 and 0.96, for HAV and GII, respectively, as compared with RTqPCR alone, without completely preventing amplification of inactivated genomes. These log_10_ reductions are similar to what has been described previously in cockles and clams inoculated with high doses of 5 min/99°C heat-inactivated HAV (1.45 and 1.53) (Moreno et al., 2015) and oysters bioaccumulated with GI and GII HuNoV and heat-processed for 15 min at 95°C (0.85 and 1.07) (Randazzo et al., 2018), confirming a similar method performance.

According to our result, PMA-viability RTqPCR method could be used to estimate whether a certain inactivation process reaches the desired performance criterion for HuNoV inactivation. Thus, heat treatments of HuNoV contaminated clams which cause reductions > 3.5 log_10_ as measured by PMA-viability RTqPCR (or > 2.5 log_10_ as measured by RTqPCR following the ISO method) may be equivalent to acquiring >3.89 log_10_ HAV inactivation. A performance criterion for a certain heat-inactivation process for HuNoV set at >3.5 log_10_ reduction, as measured by PMA-viability RTqPCR could be pursued to significantly minimize HuNoV risk. This could also be useful to test different time-temperature combinations, both during industrial and culinary processes, which could be applied to live clams from class and C production areas that have not been submitted for purification or relaying. Whether this inference could be applied to other types of shellfish and/or inactivation methods remains to be studied.

In summary, this study shows the successful use of viability PMA-RTqPCR method on complex food matrices and its potential application to evaluate whether heat-inactivation processes comply with a specific set performance criterion in terms or risk reduction. In addition, results from this study are also of value to perfect a HuNoV heat-inactivation model in shellfish samples.

## Data Availability Statement

The raw data supporting the conclusions of this article will be made available by the authors, without undue reservation, to any qualified researcher.

## Author Contributions

CF, FP-R, AS, and NB performed the experiments. RP and AB acquired funding, defined the experimental approach, revised the results, and contributed to the preparation of the first draft of the manuscript. SG defined the experimental approach, revised the results, and led the preparation of the manuscript. All authors contributed to the article and approved the submitted version.

## Conflict of Interest

The authors declare that the research was conducted in the absence of any commercial or financial relationships that could be construed as a potential conflict of interest.

## Footnotes

1 http://vassarstats.net/index.html

2 https://meatpoultryfoundation.org/content/process-lethality-spreadsheet
